# Vacuum-assisted mini-percutaneous nephrolithotomy: a new perspective in fragments clearance and intrarenal pressure control

**DOI:** 10.1007/s00345-020-03318-5

**Published:** 2020-06-26

**Authors:** Stefano Paolo Zanetti, Elena Lievore, Matteo Fontana, Matteo Turetti, Andrea Gallioli, Fabrizio Longo, Giancarlo Albo, Elisa De Lorenzis, Emanuele Montanari

**Affiliations:** 1 Department of Urology, Foundation IRCCS Ca’ Granda Ospedale Maggiore Policlinico, Via della Commenda 15, 20122 Milan, Italy; 2 Department of Urology, Foundation IRCCS Ca’ Granda Ospedale Maggiore Policlinico, Department of Clinical Sciences and Community Health, University of Milan, Milan, Italy

**Keywords:** Percutaneous nephrolithotomy, Intrarenal pressure, Urolithiasis, Endourology, Vacuum-assisted mini-PCNL

## Abstract

**Purpose:**

To describe the vacuum-assisted mini-percutaneous nephrolithotomy (vmPCNL) technique performed via the 16Ch ClearPetra sheath, to evaluate its outcomes and to analyze intrarenal pressure (IRP) fluctuations during surgery.

**Methods:**

Data from all consecutive vmPCNL procedures from September 2017 to October 2019 were prospectively collected. Data included patients’ and stones characteristics, intra and peri-operative items, post-operative complications and stone clearance. Patients undergoing vmPCNL from March to October 2019 were submitted to IRP measurement during surgery.

**Results:**

A total of 122 vmPCNL procedures were performed. Median stone volume was 1.92 cm^3^. Median operative time was 90 min and median lithotripsy and lapaxy time was 28 min. Stone clearance rate was 71.3%. Thirty-one (25.2%) patients experienced post-operative complications, seven of which were Clavien 3. Postoperative fever occurred in nine (7.4%) patients and one (0.8%) needed a transfusion. No sepsis were observed. IRPs were measured in 22 procedures. Mean IRP was 15.3 cmH_2_O and median accumulative time with IRP > 40.78 cmH_2_O (pyelovenous backflow threshold) was 28.52 sec. Maximum IRP peaks were reached during the surgical steps when aspiration is closed (mainly pyelograms), whereas during lithotripsy and suction-mediated lapaxy, the threshold of 40.78 cmH_2_O was overcome in three procedures.

**Conclusions:**

vmPCNL is a safe procedure with satisfactory stone clearance rates. Mean IRP was always lower than the threshold of pyelo-venous backflow and the accumulative time with IRP over this limit was short in most of the procedures. During lithotripsy and vacuum-mediated lapaxy, IRP rarely raised over the threshold.

## Introduction

Percutaneous nephrolithotomy (PCNL) is the standard of care for large kidney stones [1], but complications like fever and bleeding can represent a major concern [2]. To reduce the morbidity associated with this procedure, miniaturized PCNL systems were developed [3–6]. However, these systems present some limitations such as a more difficult stone fragments retrieval, a smaller visual field, longer operative times (OT) and higher intrarenal pressures (IRPs) [7, 8]. In particular, IRP higher than 30 mmHg (40.78 cmH_2_O) has been proven to cause pyelovenous backflow [9], potentially leading to infectious complications [10]. To overcome these limitations, mini-PCNL systems provided with aspirating sheaths have been introduced. The real-time suction of irrigation fluid, stone fragments and blood throughout the procedure may lower IRP, ameliorate visibility and quicken the procedure. The aim of this study is to describe the vacuum-assisted mini-PCNL (vmPCNL) technique performed using the 16 Ch Clear Petra nephrostomic sheath and to evaluate its clinical outcomes. Ultimately, we aim to analyze IRPs profile during surgery and to identify the procedural steps during which IRP may rise the most.

## Patients and methods

Data from all consecutive vmPCNL procedures performed at our academic referral stone center from September 2017 to October 2019 were prospectively collected. The indication to vmPCNL was given in all cases when PCNL was planned, except in case of large staghorn stones, for which standard PCNL (22–24 Ch) was indicated. In case of multiple stones allocated in different calyces with a significant total stone burden, for which a single standard-tract PCNL might not be surely efficacious, a multi-staged vmPCNL procedure was pre-operatively planned in order to reduce the operative time of the single procedures. Collected data concerned patients’ and stones’ characteristics, intra- and peri-operative items, post-operative complications, stone clearance and need of retreatment. Comorbidities were graded according to the Charlson Comorbidity Index [11]. Intraoperative items included number and location of the percutaneous tracts, fragments retrieval modality, lithotripsy and lapaxy time (LT) (from first laser activation to the end of fragments retrieval), exit strategy, operative time (OT) (from the beginning of ureteric catheter placement to the exit strategy) and intraoperative complications. Post-operative items included hemoglobin drop, need for transfusions, nephrostomy indwelling time and length of hospital stay. Post-operative complications were graded according to the PCNL-adjusted Clavien Score [12]. Sepsis was defined according to the Sequential [Sepsis-related] Organ Failure Assessment (SOFA) score criteria [13]. Stone clearance was defined as the absence of residual fragments larger than 4 mm at the CT scan or ultrasound (US) performed 1–3 months after surgery. All patients underwent pre-operative urographic CT scan and urine culture. In case of negative culture, one-shot antibiotic prophylaxis was administered; in case of positive culture, antibiotic targeted therapy was started 3–5 days before surgery. Stone volume was measured using the ellipsoid formula (*a* x *b* x *c* x π/6).

### Armamentarium

**Fig. 1:**
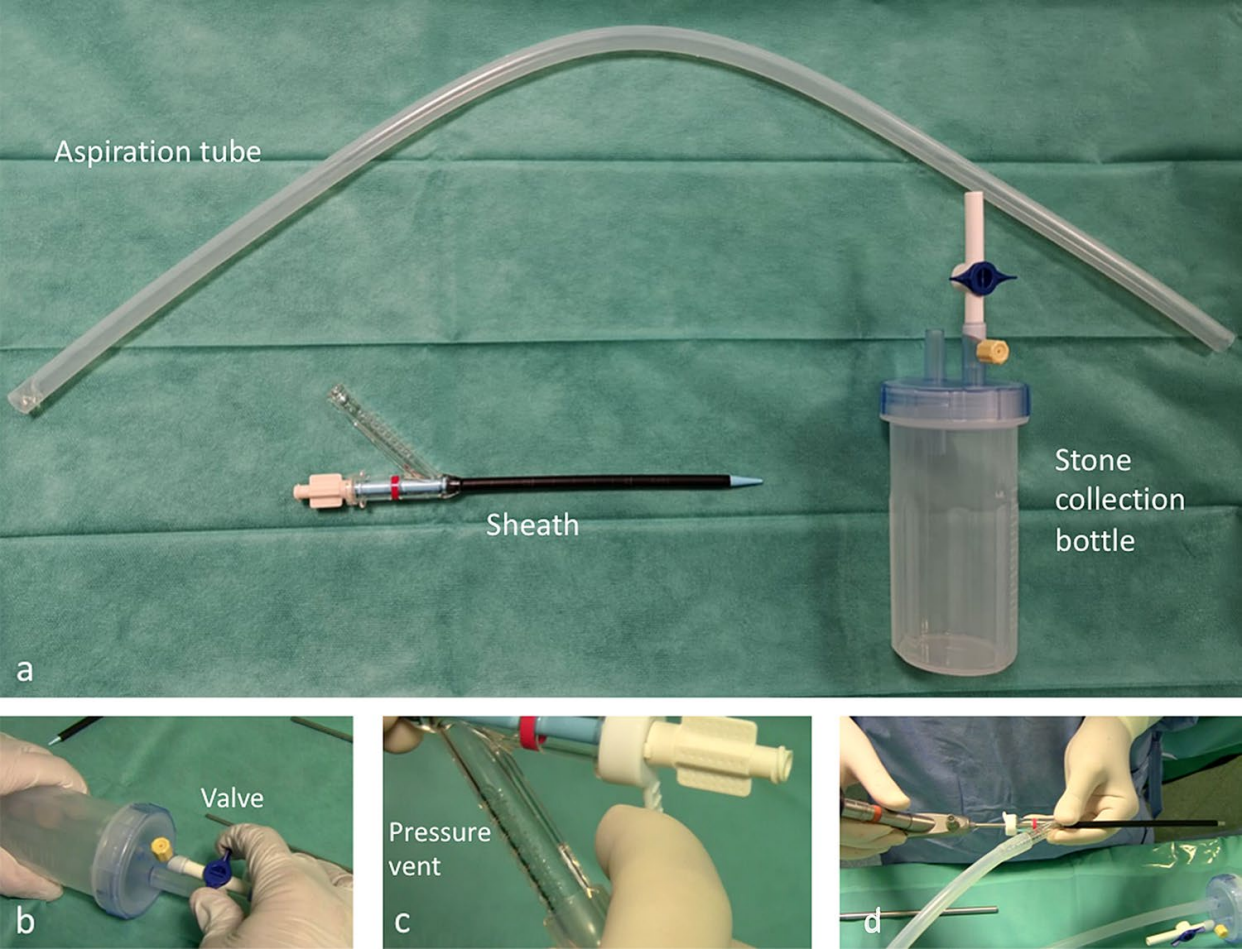
**a** The ClearPetra set (Well Lead Medical Co., Ltd., China) is composed of a Y-shaped nephrostomic sheath connected, by means of an aspiration tube, to a stone collection bottle (200 ml), which is in turn linked to the central vacuum system. **b** A valve on the stone collection bottle regulates the aspiration pressure. **c** A vent on the lateral arm of the sheath can be closed with a finger to increase aspiration pressure during lapaxy. In the meantime the nephroscope **d** is slowly drawn back until the red stripe on the sheath, thus allowing fragments to fall in the lateral arm of the sheath and in the stone collection bottle

The vmPCNL procedures were performed using a 12 Ch nephroscope (MIP set, Karl Storz) and a 16 Ch Clear Petra disposable nephrostomic sheath (Well Lead Medical Co.). This sheath is externally plugged to prevent the medium from flowing out and it is equipped with a lateral oblique arm connected to the central vacuum system (Fig. 1). This allows the continuous aspiration of stone powder and irrigation fluid beside the scope during lithotripsy. Larger fragments are retrieved by drawing back the nephroscope inside the sheath as far as the internal opening of the lateral aspiration arm, wide enough to allow the passage of stones as large as 8 mm. Aspirated stones are collected in a dedicated plastic bottle. Irrigation is provided by a saline gravity bag allocated 1.5 m above the kidney level. The aspiration pressure can be regulated throughout the procedure according to surgical needs as shown in Fig. 1, in particular it can be enhanced to ameliorate visibility in the presence of stone powder or blood and while withdrawing the nephroscope inside the sheath to extract stone fragments.

### Surgical technique

The procedure starts by placing a ureteric catheter in the renal pelvis and obtaining a retrograde pyelogram. With the patient in the semi-supine Valdivia position, renal puncture is performed under fluoroscopic/ultrasonographic control. Tract dilation is performed one-shot, using the 16 Ch Clear Petra sheath assembled with its stylet. After introducing the nephroscope in the renal cavities, the aspiration valve is switched on. Lithotripsy is performed via a 550 μm Holmium: YAG laser fiber. Stone fragments are real-time evacuated during lithotripsy through suction; a basket can be employed to catch fragments in calyces not aligned with the aspirating sheath, only reachable with a flexible scope. A nephrostomy tube is usually placed as exit strategy.

### Intrarenal pressure measurement

The subgroup of continuous patients undergoing vmPCNL from March to October 2019 were submitted to IRP measurement. After zero adjustment, IRP was measured throughout the procedures, with a frequency of 50 measurements per second, through the open-end ureteric catheter in the renal pelvis, whose external end was connected to a pressure transducer of the urodynamic machine (Medtronic, Duet^®^Multi-P). Basal IRP was recorded before renal puncture. Mean and maximum IRPs and the accumulative time with IRP > 40.78 cmH_2_O were calculated for every procedure. The procedures were split in different surgical steps to analyze IRP fluctuations along surgery.

### Statistical analysis

Data collection and analysis were performed using the statistical software SPSS 25.0.0.1 (©IBM Corp.). Mean and standard deviation (SD) and median and interquartile ranges (IQRs) were reported for continuous variables and proportions and percentages for categorical variables.

## Results

A total of 122 vmPCNL procedures were performed on 119 renal units of 115 patients. Patients’ and stones’ baseline characteristics are reported in Table 1. Seventy-four (60.7%) patients had multiple stones and the median stone volume was 1.92 cm^3^. Surgery-related variables and post-operative outcomes are shown in Table 2. Eleven procedures (9%) were performed with multiple Clear Petra tracts. A basket was employed for fragments retrieval in 44 (36.1%) procedure in addition to suction. The median OT was 90 min and the median LT was 28 min. A total of 31 (25.2%) patients experienced post-operative complications, seven out of which were Clavien 3: three cases of renal colic managed by double J placement; three cases of bleeding, one requiring bladder irrigations and two needing angioembolization; and one case of colon perforation managed by temporary colostomy. One (0.8%) patient needed a blood transfusion and nine (7.4%) patients experienced fever managed by antibiotics (Clavien 2). No case of sepsis was observed. Stone clearance was obtained in eighty-seven (71.3%) patients. Out of the 35 patients (28.7%) who did not reach the stone clear status, nine (7.4% of the total) were retreated within the first 6 month of follow-up for residual fragments larger than 10 mm: seven of them underwent a second PCNL procedure, in a pre-operatively planned multi-staged procedure, and two were submitted to retrograde intrarenal surgery. All of them resulted stone clear after the second procedure. The remaining 26 (21.3% of the total) patients had non-obstructing residual fragments smaller than 10 mm and were planned to undergo follow-up imaging.

**Table 1:** Patients’ and stones’ baseline characteristics

	Total: 122
Sex, *N*° (%)	
Male	74 (60.6)
Female	48 (39.4)
Mean age (± SD), years	55.2 (**± **14.5)
BMI, *N*° (%)	
< 18.5	5 (4.1)
18.5–24.9	60 (49.2)
25–29.9	47 (38.5)
≥ 30	10 (8.2)
Comorbidities, *N*° (%)	
Diabetes	17 (13.9)
Hypertension	32 (26.2)
Cardiovascular disease	12 (9.8)
Chronic kidney disease	3 (2.5)
Inflammatory bowel disease	5 (4.1)
Hyperparathyroidism	3 (2.5)
Haemorrhagic diathesis	2 (1.6)
Recurrent urinary tract infections	22 (18.3)
Drugs, *N*° (%)	
Anticoagulants	4 (3.3)
Antiplatelets	16 (13.1)
American Society of Anesthesiologists score, *N*° (%)	
1	22 (18)
2	79 (64,8)
3	21 (17.2)
Charlson Comorbidity Index, *N*° (%)	
0	88 (72.1)
1	19 (15.6)
2	8 (6.5)
3	3 (2.5)
4	3 (2.5)
5	1 (0.8)
Urinary tract abnormalities, *N*° (%)	6 (4,9)
Renal malrotation	2 (1.6)
Horseshoe kidney	1 (0.8)
Ectopic kidney	1 (0.8)
Duplex ureter	2 (1.6)
Single kidney, *N*° (%)	3 (2.5)
Skeletal abnormalities, *N*° (%)	2 (1.6)
Previous homolateral stone treatment, *N*° (%)	61 (50)
Stone number, *N*° (%)	
Single	48 (39.3)
Multiple	74 (60.7)
Median total stone volume (IQR), cm^3^	1.92 (1–3.1)
Median mean stone density (IQR), HU	850 (550–998)
Stone location, *N*° (%)	
Lower calyx	15 (12.3)
Middle calyx	6 (4.9)
Renal pelvis	24 (19.6)
Upper calyx	4 (3.3)
Multiple locations	73 (59.9)
Pre-operative positive urine culture, *N*° (%)	19 (15.6)
Stone composition, *N*° (%)	
Calcium Oxalate Monohydrate	38 (31.1)
Calcium Oxalate Dihydrate	26 (21.3)
Uric acid	25 (20.5)
Calcium Carbonate + Calcium Oxalate	10 (8.2)
Calcium Carbonate	7 (5.7)
Calcium Oxalate Mono + Dihydrate	5 (4.1)
Calcium Phosphate	4 (3.3)
Cystine	4 (3.3)
Struvite	3 (2.5)

**Table 2:** Surgery-related variables and post-operative outcomes

2a. Surgery-related variables	
	Total: 122
Laterality *N*° (%)	
Right	55 (45.1)
Left	67 (54.9)
Access tract number, *N*° (%)	
Single	111 (91)
Multiple	11 (9)
Access tract location, *N*° (%)	
Lower calix	84 (68.8)
Middle calix	20 (16.4)
Upper calix	9 (7.4)
Multiple locations	9 (7.4)
Median total laser energy (IQR), kJ	8.63 (3,75–16,23)
Lapaxy modality, *N*° (%)	
Clear Petra Suction	78 (63.9)
Clear Petra Suction + basket	44 (36.1)
Exit strategy, *N*° (%)	
Nephrostomy	97 (79.5)
Ureteric catheter/JJ stent	5 (4.1)
Nephrostomy + Ureteric catheter /JJ stent	20 (16.4)
Intra-operative complications, *N*° (%)	
Contrast medium blow-out	2 (1.6)
Median operative time (IQR), minutes	90 (71–120)
Median lithotripsy + lapaxy time (IQR), minutes	28 (17–45)

2b. Post-operative outcomes	
Median Hb drop (IQR) g/dL	1,5 (0.6–2.1)
Transfusions, *N*° (%)	1 (0.8)
Complications, *N*° (%)	31 (25.4)
Clavien 1	14 (11.4)
Clavien 2	10 (8.2)
Clavien 3A	4 (3.3)
Clavien 3B	3 (2.5)
Median nephrostomy time (IQR), days	3 (2–4)
Median urethral catheter time (IQR), days	1 (1–1)
Median length of hospital stay (IQR), days	4 (3–5)
Stone Clearance Rate, *N*° (%)	87 (71.3)
Retreatment, *N*° (%)	9 (7.4)

Data related to IRPs are reported in Table 3a. Twenty-two patients were included in the IRP measurement sub-cohort. The mean IRP during the procedures was 15.38 cmH_2_O. In all procedures but three, peaks over the threshold of 40.78 cmH_2_O were registered. Median accumulative time with IRP > 40.78 cmH_2_O was 28.52 s. During lithotripsy and suction-mediated lapaxy, the mean IRP was 13.29 cmH_2_O and the threshold of 40.78 cmH_2_O was overcome in three procedures. Maximum peaks were reached during pyelograms in thirteen (59%) procedures, during nephroscopy with closed aspiration in seven (32%) and during puncture in two (9%). IRP values during the different surgical steps are reported in Table 3b. Graphics representing the complete IRP profile during the 22 procedures are available as Online Resource. Two patients undergoing IRP measurement experienced Clavien 2 post-operative complications: one case of bleeding needing transfusion and one case of fever.

**Table 3:** Intrarenal pressures characteristics and fluctuations

3a. IRP characteristics and profile		
Mean basic IRP (± SD), cmH_2_O		13.19 (± 5.99)
Mean of the mean IRPs during the operations (± SD), cmH_2_O		15.38 (± 6.24)
*N*. of cases with peaks of IRP > 40.78 cmH_2_O (30 mmHg), *n*. (%)		19 (86)
N. of cases with mean IRP > 27.19 cmH_2_O (20 mmHg), *n*. (%)		0 (0)
Median accumulative time with IRP > 40.78 cmH_2_O (30 mmHg) (IQR), s		28.52 (12.5 – 60.04)
N. of cases with accumulative time (with IRP > 40.78 cmH_2_O) > 50 s, *n*. (%)		7 (31.8)
N. of cases with accumulative time (with IRP > 40.78 cmH_2_O) > 60 s, *n*. (%)		5 (22.7)
N. of cases with accumulative time (with IRP > 40.78 cmH_2_O) > 70 s, *n*. (%)		3 (13.6)

3b. Intrarenal pressure measurements step by step		
Surgical steps	Overall mean^a^ IRP (± SD), cmH_2_O	Overall max^b^ IRP (cmH_2_O)
Initial retrograde pyelography	36.14 (± 16.75)	65
Renal puncture	32.46 (± 12.49)	53,8
Percutaneous pyelography through puncture needle	37.26 (± 20.65)	102
Percutaneous tract dilation	39.10 (± 18.19)	74
Initial nephroscopy with closed aspiration, max IRP	39.03 (± 24.09)	94
Lithotripsy and simultaneous vacuum-lapaxy, mean IRP	13.29 (± 6.55)	26.53
Lithotripsy and simultaneous vacuum-lapaxy, max IRP	28.37 (± 12.26)	58
Final nephroscopy with closed aspiration, max IRP	51.15 (± 11.77)	70
Percutaneous pyelography through access sheath	53.44 (± 26.77)	115
Flexible nephroscopy after lithotripsy, max IRP	19.88 (± 8.00)	31
Nephrostomy placement	25.02 (± 15.37)	64
Final pyelography through nephrostomy tube	32.07 (± 21.24)	89

^a^Intended as the mean of the values of the single procedures for the specific surgical step

^b^Intended as the maximum value reached among the different procedures for the specific surgical step

## Discussion

To the best of our knowledge, this is the first study describing the vmPCNL technique performed with the Clear Petra nephrostomic sheath and analyzing IRPs profile during this procedure. The most described aspiration-assisted mini-PCNL system is the super-mini-PCNL (SMP) [14, 15], consisting of a fiberoptic nephroscope and a Y-shaped nephrostomic sheath connected to the aspiration. The Clear Petra sheath employs the same principles of SMP. One of the main advantages of these sets is that, while working with the aspirating sheath in close contact with the stone, the vacuum keeps the calculus in position during lithotripsy and directly attract fragments, thus preventing their scattering. In the first series of 141 patients treated with SMP [14], Zeng and colleagues reported a stone clearance rate of 90.1%, a fever rate of 11.3% and no transfusions. In our series, we obtained stone clearance in 71.3% of the patients. This result may be considered satisfactory for our real-life population, with multiple stones in 60.7% of the cases and a median total stone volume of 1.97 cm^3^. Patients treated with SMP had single stones in 88.7% of the cases and a mean diameter of 2.2 cm [14]. We recorded a median OT of 90 min, lower than the safety limit of 120 min described in the Literature [16], and a median LT of 28 min. As in the SMP series [14], we observed lower post-operative fever (7.4%) and transfusion (0.8%) rates than reported in the Literature for PCNL (10.8% and 7%, respectively) [2]. These results confirm that the principles of mini-invasiveness are respected and that short OTs and controlled IRPs could play a role in the prevention of infectious complications. This may be of extreme relevance in the present scenario characterized by increasingly frequent infections sustained by multi-drug-resistant pathogens [17, 18]. To investigate the association between elevated IRPs and infectious complications, Zhong and colleagues [19] inspected IRPs in-vivo during miniaturized PCNL. They observed that mean IRP ≥ 20 mmHg and accumulative time with IRP > 30 mmHg longer than 50 s were correlated with post-operative fever. Then, not only IRP peaks can be dangerous for kidney injuries development, but mostly the accumulative time at high pressures can be detrimental for infectious complications. Indeed, elevated IRPs and pyelo-venous backflow are associated with potential systemic absorption of bacteria often colonizing stones and subsequently contaminating the irrigation fluid during lithotripsy. In our series, mean IRP during vmPCNL procedures was 13.19 cmH_2_O and in no procedure a mean IRP > 27.19 cmH_2_O (20 mmHg) was recorded. Alsmadi and colleagues [20], who measured IRPs during SMP, registered an overall average IRP of 19.51 mmHg (26.52 cmH_2_O) and a mean IRP > 20 mmHg in 29.7% of the procedures. In both the studies, the threshold of 40.78 cmH_2_O was overpassed in most of the procedures (86% in our series and 79.7% in Alsmadi’s). However, we registered prolonged accumulative time with IRP > 40,78 cmH_2_O only in a minority of the cases (31.8%, 22.7% and 13.6% for more than 50 s, 60 s and 70 s, respectively) and the median accumulative time with IRP over the threshold was 28.52 s. Alsmadi and colleagues calculated accumulative time with IRP > 40,78 cmH_2_O longer than 50 s, 60 s and 70 s in 36%, 32.4% and 27% of the cases, respectively, and a median accumulative time with IRP over the threshold of 55 s. The slightly higher IRPs registered during SMP might be due to the use a continuous perfusion pump, that was never applied during the procedures described in the present study, in which irrigation was always provided by gravity, limiting the fluid inflow. In our series, among the cases studied for IRP fluctuations, only one patient experienced post-operative fever, thus, it was not possible to identify IRP-related predictive factors for infectious events. This patient had positive pre-operative urine culture and stone culture, treated with targeted full-course peri-operative antibiotic therapy. Mean IRP during this procedure was 13.9 cmH_2_O and the accumulative time with IRP > 40.78 cmH2O was 15 s, with a maximum peak at 60 cmH_2_O during a pyelography (Graphic n.5 in the Online Resource). IRP does not seem to have contributed to post-operative fever in this patient, but the positive cultures, even if treated, represented a risk factor. Potentially, even short times with IRP over the backflow threshold are enough to determine bacterial reabsorption in case of clearly contaminated irrigation fluids.

For what concerns the IRP fluctuations, we observed that during lithotripsy and suction-mediated lapaxy, the threshold of 40.78 cmH2O was overcome in only three procedures (13.6%) (Graphics n. 9, 11, 12 in the Online Resource), meaning that when aspiration is activated, the risk of uncontrolled pressures is low. During surgical steps, when aspiration is closed, we registered rises in IRP. Maximum pressure peeks were registered during pyelograms in 13 (59%) procedures, during nephroscopy with closed aspiration in 7 (32%) and during puncture in 2 (9%). However, the mentioned steps usually are not prolonged in duration, and do not expose the patient to long time elevated IRPs.

The main limitation of this study is the lack of a control group of mini-PCNL procedures performed without aspiration systems which could render our results more significant. One more limitation is represented by the non-uniformity of the imaging modality we adopted in the follow-up: indeed, although CT scan is much more sensitive in identifying residual fragments, in order to reduce the radiation exposure, in particular in recurrent stone formers, we routinely perform US after uneventful procedures performed for small single stones, in which the chance of residual fragments is low. In case of residual fragments at US, a CT scan is performed to plan a retreatment.

## Conclusions

VmPCNL via the 16 Ch Clear Petra sheath is characterized by a good safety profile and satisfactory stone clearance rates. The most interesting features of this technique are the easy suction-mediated stone fragments removal, the low complications rate and the favorable IRP profile. In particular, in our series, the mean IRP during surgery was always lower than the threshold of pyelo-venous backflow and the accumulative time with IRP over this limit was very short in most of the procedures. The maximal IRP peaks were registered during the surgical steps when aspiration is closed and mostly during pyelograms, whereas, during lithotripsy and vacuum-mediated lapaxy, IRP rarely raised over the threshold.
